# Discriminating Between Fallers and Non-Fallers Using Kinematic Data from the Heel2Toe™ Wearable Sensor

**DOI:** 10.3390/s26051716

**Published:** 2026-03-09

**Authors:** Nancy E. Mayo, Ahmed Abou-Sharkh, Helen Dawes, Sarah J. Donkers, Chelsia Gillis, Krista Goulding, Edward Hill, Kedar Mate, Yosuke Tomita

**Affiliations:** 1 Department of Medicine, McGill University, Montreal, QC H3A 0G4, Canada; 2 Center for Outcomes Research and Evaluation, Research Institute—McGill University Health Center, Montreal, QC H3A 0G4, Canada; 3 PhysioBiometrics Inc., Montreal, QC H3A 0G4, Canada; ahmed@physiobiometrics.com (A.A.-S.); h.dawes@exeter.ac.uk (H.D.); ted@physiobiometrics.com (E.H.); kedar.mate@mail.mcgill.ca (K.M.); 4 Faculty of Health Sciences, University of Exeter, Exeter EX4 4PY, UK; 5 School of Rehabilitation Science, University of Saskatchewan, Saskatoon, SK S7N 2Z4, Canada; sarah.donkers@usask.ca; 6 School of Human Nutrition, McGill University, Montreal, QC H3A 0G4, Canada; chelsia.gillis@mcgill.ca; 7 Department of Orthopaedics, Mayo Clinic College of Medicine, Pheonix, AZ 85054, USA; goulding.krista@mayo.edu; 8 Faculty of Medicine and Health Sciences, McGill University, Montreal, QC H3A 0G4, Canada; 9 Department of Physical Therapy, Takasaki University of Health and Welfare, Takasaki 370-0033, Japan; tomita-y@takasaki-u.ac.jp

**Keywords:** gait, kinematics, falls, wearable, sensor, digital

## Abstract

Most falls occur while walking, making gait quality a logical therapeutic target. Many temporo-spatial variables have been implicated in increased fall risk, but these are dependent upon kinematic parameters of the joints involved in the gait cycle. The widespread availability of wearable sensors has made the acquisition of kinematic data feasible, and those related to the ankle are most relevant, as they relate most closely to causes of falls, trips, slips, and mis-steps. The purpose of this study is to estimate the extent to which measures of ankle angular velocity (AV) during walking are associated with falls. This is a comparative study of ankle AV metrics between people who have or have not experienced a fall in the past year. Data came from experimental use of the Heel2Toe™ sensor in a variety of settings, including demonstrations and clinical research studies. The sample comprised 387 participants, of whom 68 (17.6%) self-reported falling in the past year. Logistic regression with a natural cubic spline with 3 degrees of freedom identified AV of the angle at heel strike to discriminate between fallers and non-fallers, and the regression parameters were used to propose an algorithm to estimate fall risk. Applying the algorithm to the existing data yielded a range of probabilities from 0.0480 to 0.7245 depending on age of the person assessed. Further testing of this algorithm in different samples is warranted.

## 1. Introduction

There is considerable clinical and research interest in risk factors for falls [1,2] and fall risk assessment. Based on these risk factors, a number of fall risk algorithms have been developed, put into use, and systematically summarized [3,4,5].

The methods to identify risk factors and develop these algorithms have varied. One method has been to identify fallers, usually through self-reports of falls in the past, and estimate the extent to which current values on a set of candidate variables discriminate between fallers and non-fallers [6,7,8,9,10]. This method assumes participant recall is accurate, and that the fall itself did not change the values on the variables of interest, a reverse-causality phenomenon.

A second, and by far the strongest, method is to assemble a representative sample of the target population, assess all at study entry, follow participants into the future to ascertain the fall episodes for all, and then link variables at study entry to fall occurrence or occurrences. A cohort study provides the most accurate estimates to develop an algorithm when there is complete follow-up of the cohort [11,12,13,14,15].

A third method is, within a representative cohort, to identify those who fall (cases), match these cases to one or more controls who have not fallen, and look at historical data to identify factors predicting fall status, a case–control study [16,17].

The candidate variables fall into two groups: intrinsic (related to the person), and extrinsic, (related to the environment) [18,19]. Within the intrinsic group, a further distinction is made between modifiable and non-modifiable variables, and within the non-modifiable ones, there are factors that pre-dispose someone to fall, like medications that might alter blood pressure or vigilance, and factors related to the circumstances of the fall. In a 2022 systematic review of 31 studies involving 70,868 community seniors, the strongest intrinsic risk factors for falls were mainly non-modifiable factors, including dementia (2.01, 95% CI: 1.41–2.86), age (1.15, 95% CI: 1.09–1.22), female gender (1.52, 95% CI: 1.27–1.81), fear of falling (2.82, 95% CI: 1.68–4.74), history of falls (3.22, 95% CI: 1.98–5.23), and vision unclear (1.56, 95% CI: 1.29–1.89). The strongest modifiable factors were depression (1.23, 95% CI: 1.10–1.37), and balance disorder (3.00, 95% CI: 2.05–4.39) [1].

Of the modifiable factors contributing to falls, the greatest interest is in those related to gait pattern, as most falls occur when someone is upright and walking, [7,20,21,22,23,24], and balance and gait are amenable to intervention [25,26,27,28].

Many features of gait, which is the manner of walking, have been implicated in falls, and these are classified as temporo-spatial, kinetic, and kinematic gait parameters. Temporo-spatial gait parameters refer to measures of distance, time, or distance by time such as step and stride length, cadence, gait speed, step and stride time, time and duration of swing, and stance phases of gait [29]. Kinematic parameters relate to range of motion at different joints and body segments during walking, and kinetic parameters relate to ground reaction forces generated during walking [30,31]. Kinematic and kinetic data are generated from technologically advanced systems such as motion capture systems, instrumented walkways, and force plates [32]. However, with the increasing number and sophistication of wearable sensors, it is now possible to measure these gait quality parameters outside of the laboratory without the expertise, cost, and infrastructure associated with these complex systems, and then relate these data to health events, such as falls.

Several temporo-spatial variables have been related to falls, including gait speed, stride length, and single leg stance [11], and these metrics are readily available from wearable sensors. Kinematic parameters are less easily obtained, but the widespread availability of wearable sensors has made the acquisition of kinematic data feasible [33]. The kinematic parameters that have been linked to falls have been measured during the different phases of the gait cycle (heel strike, foot flat, push-off, swing). These include metrics related to variability in time spent in the different phases of gait, leg velocities, ankle angle velocities, and ankle angles [34,35,36,37,38,39].

These studies, and others deriving metrics or non-linear dynamic measures from sensor outputs from different body segments [40], provide a list of potential kinematic variables that discriminate between fallers and non-fallers, but do not provide an algorithm to predict an individual’s risk of falling. This is an important parameter because reducing fall risk can be a targeted outcome, similarly to cardiovascular risk [41,42,43].

Angular velocities of the ankle during the gait cycle are highly relevant metrics, as they relate most closely to causes of falls, trips, slips, and mis-steps [8,44]. The components of the normal gait pattern have described showing the sequential components, starting with heel strike; subsequent phases are foot flat, push-off, and swing [45,46,47].

**Figure 1:**
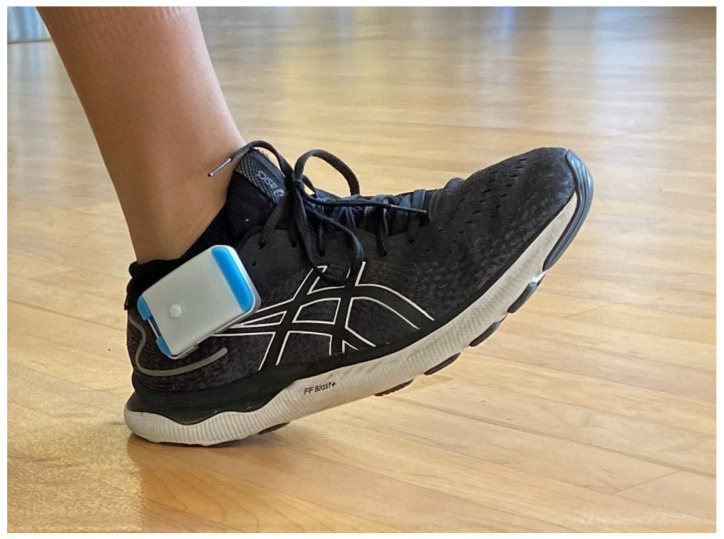
Heel2Toe™ sensor on the right shoe.

A new sensor, the Heel2Toe™ sensor, developed by PhysioBiometrics Inc., was developed specifically to measure ankle angular velocities during walking, as human bipedal gait is characterized by a heel-to-toe pattern [45], and losing that pattern has been associated with falls [48,49]. The Heel2Toe sensor, shown in Figure 1, is one of a new generation of smart wearables that, in addition to providing rich data to accurately measure gait, also provides feedback for a gait pattern that starts with a strong heel strike, providing therapy in every step [50].

The purpose of this study is to estimate the extent to which measures of ankle angular velocity during walking are associated with falls. This information would be used to quantify fall risk among gait vulnerable people and assess the extent to which interventions to improve gait quality would reduce this risk.

## 2. Materials and Methods

This is a comparative study of ankle angular velocity measures between people who have or have not experienced a fall in the past year.

### 2.1. Source of the Data

The data available for this study came from experimental use of the Heel2Toe sensor (PhysioBiometrics Inc., Montreal, QC, Canada) in a variety of settings, including demonstrations and clinical research studies. Data collected during demonstrations of the sensor were not originally acquired for research purposes, and, therefore, permission to use these data for research was granted by the Institutional Review Board of McGill University (A09-M51-23B), as no personal data was collected that could be used to identify the individual. All the clinical research studies using the sensor had ethical approval from their local research review boards, and no identifying information was available to the research team associated with this project.

### 2.2. Sample

Only participants for whom it was possible to identify their age, disability status, and fall status were included. Excluded were participants with less than 10 steps during a walking session.

### 2.3. Measurement

Age was classified as 75 years and older, or not. As the data came from many different sources, including testing situations and clinical studies, age was not consistently reported. For the data arising from testing, no personal data was collected, and testers were asked to retrospectively assign age. Many were obviously known to be young, but, for others, the testers felt they could accurately assign people to above or below 75 years of age. The clinical studies recorded age, but our agreement with ethics was that we would not ask for any information that could potentially identify a specific individual, so we asked researchers who donated data to classify their participants at this cut-point. The most common cut-points identified as increasing fall risk are 65, 75 and 80 years. References [51,52] and evidence show a dramatic drop in walking activities after the age of 75 [53], supporting this decision.

Disability status was a binary variable based on diagnosis or use of a walking aid. Fall status (yes or no in the past year) was ascertained as part of the clinical research studies and also queried during demonstrations for people with disabilities. Fall frequency was not available, as some clinical studies had more than 1 fall in the past year as an exclusion criterion. Data on sex was not consistently available. From the available descriptive information, 5 strata were formed. One stratum comprised people <75, without a disability, and non-fallers. This stratum served as a reference group. Another stratum comprised the fallers. The other three strata were comprised of non-fallers who were either older, with a disability, or both.

The Heel2Toe sensor attaches to the side of shoe and detects motion of the ankle during the gait cycle. The hardware for the Heel2Toe sensor consists of a microprocessor (MCU) running at 150 million instructions per second, an accelerometer, gyroscope, and magnetometer (collectively called an Inertial Measurement Unit (IMU)), each with 3 degrees of freedom running at 100 Hertz, or samples per second.

Metrics available from the Heel2Toe sensor for each step in a walking session are: angular velocity (AV) during heel strike, push-off, and foot swing. These metrics are averaged over steps taken during each walking session, and coefficient of variation (CV) is also calculated. Also available from the sensor is the time for each part of the gait cycle, which can be used to calculate cadence and single leg stance.

Angular velocity of ankle rotation is extracted from the gyroscope (Z axis), clockwise movement is recorded as negative values (the more negative indicates higher velocity), movement anti-clockwise is recorded as positive values. Movement of the foot from initial heel strike to foot flat is a clockwise movement, as is foot flat to push-off, so both heel strike AV and push-off AV have negative values. To clear the foot during swing phase, the foot must rotate counter-clockwise, and, hence, AV for foot clearance has a positive value.

### 2.4. Statistical Analysis

Each walking session comprises multiple steps; therefore, each gait metric was presented as the mean and standard deviation (SD) over all steps, which was used to derive the CV over the steps of the walking session. The sample was described using the mean and the SD of the mean, and the mean and SD of the CV (presented as absolute value), as well as the range. Spearman correlations were conducted for all pairs of kinematic variables. A linear model was used to regress temporo-spatial and kinematic variables on the variable representing the 5 strata, with the stratum representing non-fallers, <75 years, and without disability as the referent group.

As the gait cycle is set up from heel strike, our a priori model was to regress fall status on heel strike and age. The other components of the gait cycle were considered as the consequences of the magnitude of the heel strike. In the binary outcome, fall status was regressed on heel strike AV using logistic regression. As heel strike AV is a continuous variable, non-linear effects were modeled with a natural cubic spline (ns) with 3 degrees of freedom (df). Also tested were b-spline and penalized spline models, and ns with 6, 4, and 2 df. As age and disability status were related, and, as the effect of disability on kinematic variables and falls is well known, the model considered age only.

A model with all available kinematic and temporo-spatial models was also assessed to substantiate the independent effect of heel strike. Model reduction was based on difference in deviance and the Akaike Information Criterion (AIC).

The ns spline model was examined for a logical cut-point for estimating risk. Odds ratios at different cut-points along the continuum were generated to aid in identifying the inflection point [54]. A segmented regression model was also used for this purpose, although mis-specified, owing to fitting straight lines through a curve. Also calculated were fall classification accuracy parameters of sensitivity and specificity at different values of heel strike Avs, along with a receiver operating characteristic curve.

The regression coefficients from a cut-point stratified analysis were used to create the algorithm to produce estimates of individual fall risk at different values of heel strike.

### 2.5. Power

As this study took advantage of existing data, power for a fixed sample size is the relevant estimate to consider. There were 68 fallers and 319 non-fallers; thus, even with the conservative rule-of-thumb of 10 events per variable, this study has adequate power for testing 6 to 7 predictors [55].

## 3. Results

### Characteristics of the Sample

The sample comprised data on 387 participants who were assessed using the Heel2toe sensor; 68 (17.6%) of the participants self-reported falling in the past year. Of the sample, 240 (62.0%) were <75 years of age, and 147 (38.0%) were 75+ years; 148 (39.2%) were classified as having a disability: 29 with Parkinson’s Disease, 12 with Multiple Sclerosis, 33 with orthopedic conditions, 18 with soft tissue sarcoma, and 2 with other disability situations; 53 needed a walking aid. Table 1 presents temporo-spatial and kinematic characteristics of the sample according to five strata. In comparison to the reference stratum (n = 205) of non-fallers, <75 years, and without disability, the other samples all had less optimal values. The variables that differed between these strata and the reference sample are bolded and indicated by the inclusion of a *p*-value when it was <0.05. The variables that consistently distinguished all other strata from the referent strata were metrics related to AV.

Table 2 presents Spearman correlation coefficients among the nine kinematic gait parameters tested in this study for their relationships with fall status. As these variables are part of the same construct, correlations of 0.8 or more are considered strong, and 0.5 to 0.8 are considered moderate. These are highlighted in the deepest blue. In lighter shades of blue are correlations of 0.4 and correlations of 0.3. The strongest correlations are among the measures of AV.

**Figure 2:**
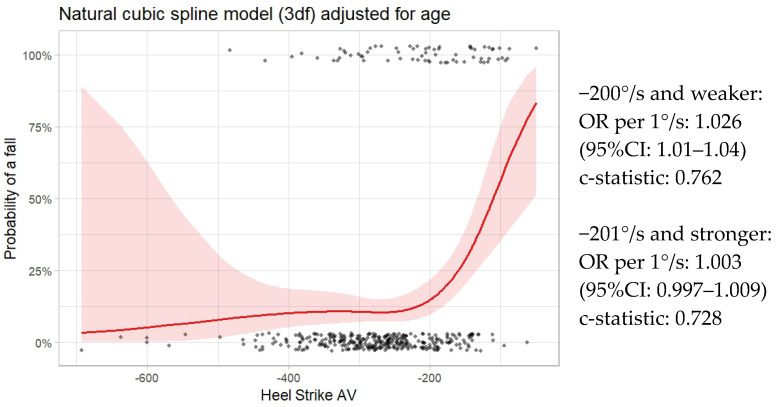
Relationship of heel strike AV to faller status adjusted for age (red line with 95% CI). Dots along the bottom (top) of the graph show the locations along the x-axis for non-fallers (fallers).

The results of the logistic model with a 3df ns spline for heel strike and adjusted for age are shown in Figure 2. Values for heel strike AV are presented on the x-axis, and probability of a fall expressed as a percent is on the y-axis. The dots at the bottom of the graph indicate the values of non-fallers along the x-axis, and those at the top of graph indicate the locations of the fallers. The graph clearly shows the non-linear relationship between heel strike AV and probability of falls. The shaded areas around the line show the 95% CI. As can be seen, there is more confidence when values of heel strike AV are more than −450°/s. Also evident is that the relationship changes considerably when values of heel strike AV approach −200°/s. The predictive accuracy of this model, as illustrated by the c-statistic, is 0.7914, considered very good.

The segmented regression model suggested a cut-point of −156°/s. (95% CI: −196 to −116; c-statistic: 0.7737). Figure 2 shows that a cut-point to the left of that value is more representative of the graph.

Figure 2 shows the risk equation for each of the two segments of the sample, one to the left (stronger) and one to the right (weaker) of the −200°/s cut-point. For the left side of the cut-point, there was a non-significant relationship between faller status and heel strike AV with more optimal values (OR per 1°/s: 1.003; 95% CI: 0.997–1.009). For the right side of the cut-point, with less optimal values, there was a strong association (OR per 1°/s: 1.026; 95% CI: 1.01–1.04) with fall risk. These values appear small because the units are per 1°/s. If the regression parameters are estimated per 50°/s, the OR are 1.17 and 3.45 for the two segments, respectively. For the less optimal segment (≥−200°/s), for every 50 degrees that heel strike is less optimal, the odds of being a faller is increased by a factor of 3.45.

The full model, with all kinematic and temporo-spatial gait parameters, did not show better fit to the model than the hypothesized one with heel strike and age (AIC 309 and 307 for full and hypothesized, respectively).

Table 3 presents the predicted probability of a fall for people with heel strike AV stronger (more negative) than −200°/s and for people with heel strike AV weaker (closer to 0) than −200°/s, according to age category. The regression equations for each of these four strata are also presented.

While cadence was associated with faller status overall, its effect when the data set was stratified by magnitude of heel strike AV was not significant, and its effect on the predicted probability estimates was negligible. The lowest risk of falling was observed for cadences between 80 and 100 steps per minute, nadir 90 steps per minute.

**Figure 3:**
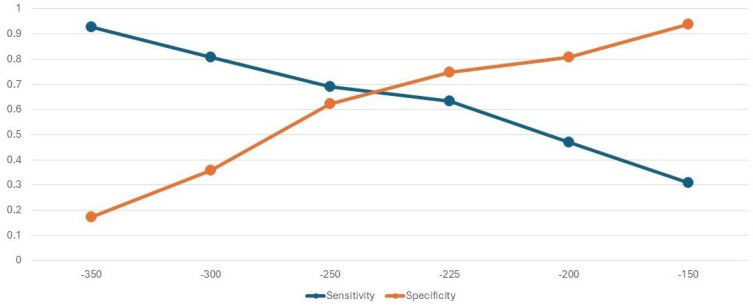
Sensitivity and specificity for discriminating fallers and non-fallers according to angular velocity of heel strike.

Figure 3 shows the sensitivities and specificities for discriminating fallers from non-fallers at different values of heel strike AV. The point where both parameters are optimized is close to −250°/s. The results of the ROC curve analysis suggest an optimal cut-point of −227°/s (AUC: 0.7029; moderate classification performance). Depending on the context of use, different cut-points could be considered. For example, at −300°/s, over 80% of people were correctly classified as fallers, and an individual less than 75 years of age would have a predicted (future) risk of 6.4%, but 28% if ≥75 years of age (see Table 3). This would be an appropriate cut-point for recommending interventions to reduce fall risk. However, at this cut-point, there is a danger in flooding fall intervention programs with people classified as high risk who are, in fact, not.

**Figure 4:**
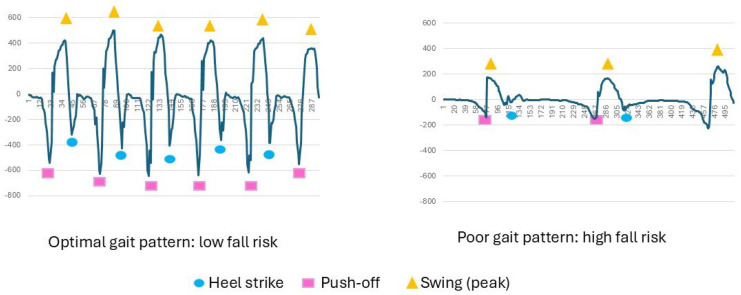
Examples of optimal and poor gait patterns.

Figure 4 shows the gait pattern of two individuals, extracted from the data from the z-axis of the gyroscope of the Heel2Toe™ sensor positioned on the outside of the right shoe just under the lateral malleolus; one person has an optimal gait pattern and a very low risk of fall, and the second person shows with a very poor gait pattern and a high risk of falls.

## 4. Discussion

The results of this analysis indicate that starting the gait cycle with a strong heel strike has the potential to reduce fall risk. One reason for this is that a strong heel strike sets up the other phases of the gait, in particular, optimal foot clearance [44]. Foot scuffing has been implicated in falls, but the strategy to increase foot clearance is to emphasize a heel-to-toe gait pattern [48,56].

AV at push-off has previously been found to be associated with falls in a small sample of 163 seniors of mean age 82.6 years [36]. But few other studies have measured AV at the ankle. Some studies measured the degree of dorsi-flexion at the ankle during initial contact. One study measured AV, but the sensor was placed at mid shank, representing leg movement rather than ankle movement [37,39]. A number of systematic reviews describe the potential utility of sensor-derived kinematic data from sensors placed on the lower limb or on the lower back for the assessment of fall risk, but no current fall risk prediction algorithms have been proposed [40,57,58].

Our values on gait parameters are somewhat different than those reported by Beauchet et al. for a healthy sample of people 65+ years from two consortiums (n = 954). Using the GAITrite^®^ system, mean stride time for people 75+ was 1.15 s, compared with >1.30 s in our samples (see Table 1). Similarly, Beauchet et al. reported single leg stance values of 0.41 s, whereas, in our sample, the value over the full sample was lower, approximately 0.31 s. This likely reflects that 50% of our sample had a disabling health condition or a history of falls.

The results of this analysis indicate that starting the gait cycle with a strong heel strike has the potential to reduce fall risk, indicating heel strike as a therapeutic target for fall prevention. Nevertheless, the target cut-point is not definite and needs further study. Our analyses point to −200°/s as a threshold value where fall risk changes. Analyses of sensitivity and specificity (see Figure 3) show moderate classification performance, but can be used to guide selection criteria for entry into fall prevention programs.

This study is limited in that fall status was modeled and not fall incidence. Thus, this algorithm needs to undergo further testing. In addition, the data came from data accumulated from research projects where the sensor was used and, hence, the amount of data available was varied, as a result age was inferred for some and dichotomized for all. The sample was also not selected to be representative of any particular segment of the population, but was obtained from people using the sensor in real-world settings. Future studies are needed to test the robustness of this algorithm in different populations and walking contexts.

The results presented here open up additional avenues of scientific enquiry and implementation. The Heel2Toe™ sensor was developed primarily as a gait training device, as its main functionality is to provide auditory feedback for a good step, one in which the AV at heel strike surpasses a specific threshold. This reward–feedback loop promotes neuroplasticity, likely through the dopamine system [50]. While there are many effective exercise programs targeting balance [59], few target the elements needed for an optimal gait pattern: knowledge, strength and mobility of muscles and joints of the lower limb, core strength, all facets of balance (static, dynamic, anticipatory, and reactive), and coached training [60,61]. Paradoxically, indoor or outdoor walking programs have low evidence for efficacy and could even be harmful [61], and we hypothesize this negative effect could be because a sub-optimal gait pattern can increase scuffing, tripping, or mis-stepping, particularly as the person fatigues [62]. In addition, the role of sarcopenia in gait failure could be mediated through optimization of macro- and micronutrients known to be needed for muscle power and coordination [63]. Preventing falls is a complex goal and needs a multi-modal approach, as each element is likely to increase the effectiveness of the other elements.

## 5. Conclusions

Average angular velocity at heel strike over a walking session of a minimum of 10 steps was the key kinematic variable discriminating between fallers and non-fallers and, together [46] with age (≥75 vs. <75 years), was used to propose an algorithm to estimate individual fall risk. Classification accuracy metrics at different cut-points can be used for program planning depending on the context. Further validation of this risk model is needed.

## Figures and Tables

**Table 1:** Average values for temporo-spatial and kinematic parameters calculated over a walking session according to strata defined by fall status, age, and disability.

Variable	Mean (SD)	Range	*p*-Value (<0.05) vs. Referent
**Stratum 1: Reference sample of non-fallers, <75 years, no disability (n = 205)**			
Number of steps	68.7 (132.3)	10.0–1724.0	Referent
Stride time (s)	1.27 (0.28)	0.62 –2.44	Referent
Cadence (steps/min)	98.0 (19.2)	49.3–193.3	Referent
Single leg stance (s)	0.31 (0.03)	0.16–0.47	Referent
Heel strike AV (−°/s)	−289.12 (88.4)	−692.89–−130.78	Referent
|Heel strike CV|	0.31 (0.15)	0.05–0.88	Referent
Push-off AV (−°/s)	−393.2 (134.4)	−911.9–−55.3	Referent
|Push-off CV|	0.29 (0.23)	0.02–1.44	Referent
Foot swing AV (°/s)	362.9 (85.7)	175.5–719.2	Referent
|Foot swing CV|	0.16 (0.11)	0.03–0.61	Referent
**Stratum 2: Non-faller, <75, with disability (n = 35)**			
Number of steps	171.7 (130.5)	17.0–562.0	
Stride time (s)	1.33 (0.39)	0.87–2.59	
Cadence (steps/min)	96.3 (22.0)	46.4–137.2	
Single leg stance (s)	0.32 (0.03)	0.25–0.36	
Heel strike AV (−°/s)	−297.3 (84.6)	−436.8–−147.0	
|Heel strike CV|	0.28 (0.12)	0.10–0.61	
Push-off AV (−°/s)	−360.8 (153.4)	−629.4–−4.7	
**|Push-off CV|**	**0.47 (1.38)**	**0.05–8.37**	**0.0460**
**Foot swing AV (°/s)**	**344.5 (109.6)**	**165.9–588.2**	**<0.0001**
|Foot swing CV|	0.14 (0.06)	0.06–0.31	
**Stratum 3: Non-faller, 75+, no disability (n-9)**			
Number of steps	95.0 (80.2)	18.0–254.0	
Stride time (s)	1.45 (0.39)	1.05–2.26	
Cadence (steps/min)	87.2 (19.6)	53.1–114.4	
Single leg stance (s)	0.31 (0.05)	0.20–0.39	
Heel strike AV	−255.5 (122.6)	−496.4–−133.8	
|Heel strike CV|	0.39 (0.14)	0.19–0.64	
**Push-off AV**	**−267.7 (124.6)**	**−507.7–−97.2**	**0.0110**
|Push-off CV|	0.48 (0.33)	0.10–1.19	
**Foot swing AV**	**293.1 (115.4)**	**156.6–549.6**	**0.0256**
|Foot swing CV|	0.21 (0.12)	0.11–0.51	
**Stratum 4: Non-faller, 75+, with disability (n = 70)**			
Number of steps	95.9 (85.0)	13.0–502.0	
Stride time (s)	1.32 (0.35)	0.92–2.62	
Cadence (steps/min)	95.4 (18.9)	45.8–130.5	
**Single leg stance (s)**	**0.30 (0.03)**	**0.21–0.39**	**0.0488**
**Heel strike AV**	**−241.6 (82.0)**	**−463.7–−62.3**	**0.0001**
|Heel strike CV|	0.32 (0.16)	0.09–1.00	
**Push-off AV**	**−311.2 (148.1)**	**−721.6–−64.6**	**<0.0001**
|Push-off CV|	0.29 (0.28)	0.05–1.77	
**Foot swing AV**	**313.2 (93.0)**	**136.7–657.2**	**0.0001**
|Foot swing CV|	0.16 (0.09)	0.04–0.56	
**Stratum: Faller (n = 68)**			
Age 75+	29 (63.2%)		
Disability	43 (91.2%)		
Number of steps	111.7 (102.4)	10.0–395.0	
**Stride time (s)**	**1.41 (0.42)**	**0.63–2.60**	**0.0052**
Cadence (steps/min)	92.7 (26.8)	46.1–192.0	
Single leg stance (s)	0.30 (0.04)	0.20–0.44	
**Heel strike AV**	**−213.0 (92.9)**	**−483.2–−49.0**	**<0.0001**
**|Heel strike CV|**	**0.35 (0.16)**	**0.10–0.80**	**0.0333**
**Push-off AV**	**−286.7 (165.2)**	**−652.5–−20.7**	**<0.0001**
|Push-off CV|	0.40 (0.45)	0.05–2.83	
**Foot swing AV**	**283.2 (93.7)**	**116.4–500.2**	**<0.0001**
|Foot swing CV|	0.14 (0.08)	0.04–0.42	

|absolute value|; (−°/s) higher values are closer to 0 and indicate a less optimal value for heel strike and push-off; (°/s) higher values indicate more optimal foot swing. Variables that are in bold differ from referent values with *p*-value <0.05.

**Table 2:** Spearman correlations among kinematic gait parameters.

		Cadence	Single Leg Stance	Heel Strike AV	Heel Strike CV	Push-Off AV	Push-Off CV	Foot Swing AV	Foot Swing CV
**Step time (s)**		*−1.000*	*−0.154*	*0.319*	*−0.069*	*0.441*	*−0.502*	*−0.332*	*0.203*
	**Cadence**		*0.154*	*−0.319*	*0.068*	*−0.441*	*0.502*	*0.332*	*−0.203*
		**Single leg stance**		*−0.348*	*0.228*	*−0.459*	*0.225*	*0.416*	*−0.271*
			**Heel strike AV**		*−0.297*	*0.570*	*−0.263*	*−0.784*	*0.063*
				**Heel strike CV**		*−0.256*	*0.447*	*0.274*	*−0.458*
					**Push-off AV**		*−0.574*	*−0.720*	*0.423*
						**Push-off CV**		*0.335*	*−0.671*
							**Foot swing AV**		*−0.186*

**Table 3:** Probability of fall for people with Heel Strike AV stronger and weaker than −200°/s according to category of age.

Age (Years)	Heel Strike AV (°/s)	Predicted Probability of a Fall
Heel strike AV stronger than −201°/s		
Predicted probability = 1/(1 + EXP(−(−1.7441 + 0.00311*HeelStrikeAV + 0*Age)))		
<75	−201	0.0858
<75	−225	0.0799
<75	−250	0.0744
<75	−275	0.0692
<75	−300	0.0643
<75	−325	0.0598
<75	−350	0.0556
<75	−375	0.0516
<75	−400	0.0480
Predicted probability = 1/(1 + EXP(−(−1.7441 + 0.00311*HeelStrikeAV + 1.7339*Age)))		
≥75	−201	0.3463
≥75	−225	0.3296
≥75	−250	0.3127
≥75	−275	0.2962
≥75	−300	0.2803
≥75	−325	0.2648
≥75	−350	0.2500
≥75	−375	0.2357
≥75	−400	0.2220
Heel strike AV −200°/s or weaker		
Predicted probability = 1/(1 + EXP(−(2.7323 + 0.0253*HeelStrike AV + 0*Age)))		
<75	−200	0.0889
<75	−175	0.1551
<75	−150	0.2568
<75	−125	0.3941
<75	−100	0.5504
<75	−200	0.1739
<75	−175	0.2838
<75	−150	0.4272
<75	−125	0.5840
Predicted probability = 1/(1 + EXP(−(2.7323 + 0.0253*HeelStrike AV + 0.7693*Age)))		
≥75	−200	0.1739
≥75	−175	0.2838
≥75	−150	0.4272
≥75	−125	0.5840
≥75	−100	0.7254

## Data Availability

These data are not available, but the algorithm is given in the paper. We did not seek ethical approval for data sharing, as the data have been donated from other projects.
